# Contextualist model evaluation: models in financial economics and index funds

**DOI:** 10.1007/s13194-022-00506-5

**Published:** 2023-01-21

**Authors:** Melissa Vergara-Fernández, Conrad Heilmann, Marta Szymanowska

**Affiliations:** 1 Erasmus Institute for Philosophy and Economics (EIPE), Erasmus School of Philosophy, Erasmus University Rotterdam, Rotterdam, Netherlands; 2 Department of Finance, Rotterdam School of Management, Erasmus University Rotterdam, Rotterdam, Netherlands

**Keywords:** Models, Model evaluation, Performativity, Reactivity, Finance, CAPM

## Abstract

Philosophers of science typically focus on the epistemic performance of scientific models when evaluating them. Analysing the effects that models may have on the world has typically been the purview of sociologists of science. We argue that the reactive (or “performative”) effects of models should also figure in model evaluations by philosophers of science. We provide a detailed analysis of how models in financial economics created the impetus for the growing importance of the phenomenon of “passive investing” in financial markets. Considering this case motivates the position that we call contextualism about model evaluation, or *model contextualism* for short. Model contextualism encompasses standard analyses of the epistemic performance of the model, but also includes their reactive aspects. It entails identifying the *epistemic and contextual import of the model*, the ways in which a model can engender change in the world (which we call the *channels of transmission*), and the *interactions between the epistemic and reactive import of a model*.

## Introduction

Scientific models have been important for philosophers of science to study because of their epistemic import; their capacity to allow model users to learn new facts about phenomena. And so, traditionally, philosophers have appraised models on the basis of their epistemic contributions (Claveau & Vergara Fernández, 2015); their explanatoriness—e.g. Aydinonat, 2018; Batterman & Rice, 2014; Hindriks, 2008; Hochstein, 2017; Lisciandra & Korbmacher, 2021; Marchionni, 2017; Reiss, 2012; Verreault-Julien, 2018—or capacity to convey understanding—e.g. Knuuttila & Merz, 2009; Reutlinger et al., 2018; Verreault-Julien, 2017; Weisberg, 2013; Ylikoski & Aydinonat, 2014. Much of the philosophical work has thus been concentrated in both elucidating in virtue of what models have their epistemic import—e.g., their ontology or the representational relation in which they stand with their target—and the reach of their import—e.g., whether they are explanatory and/or yield (mere) understanding. In general, the focus has been on why and to what extent are models able to capture (represent) the world in order to understand it.

Much less attention has been devoted by philosophers to study the way in which models are capable of shaping the world.^1^ Instead, this work has been mostly taken up by sociology of science scholars, who have found in finance a ubiquitous instance of how science can shape reality—they call it “performativity”. Famously, sociologist of science Donald MacKenzie (MacKenzie et al., 2007; MacKenzie, 2006a, 2006b) has been adamant in claiming that economics is performative, meaning that “an effect of the use in practice of an aspect of economics [a theory, model, etc.] is to make economic processes more like their depiction by economics” (2006b, p. 30).^2^ Particularly with respect to economics, some philosophers have now taken this cue from sociologists and started to explore this aspect more generally—e.g. Boldyrev and Svetlova (2016). Other recent work is Boldyrev and Ushakov (2016), who argue that models can be built with the purpose of shaping the world, as in the case of the models of general equilibrium built by Hurwicz, and that this ought to be accounted for in our philosophical accounts of models; Tee (2019) who argues that, in addition to the traditional criterion of model-world representation, at least some models could be evaluated by their “constructive” capacity; and van Basshuysen et al. (2021), who, in the context of the Covid-19 pandemic suggest ways in which predictive epidemiological models affect their targets and raises important questions about the implications this has for accounts of models.

In this article, we suggest also that we should reconsider how to appraise cases in which the significance of models is determined, at least partly, by their success in making aspects of reality resemble the model. But more than simply sympathise with previous contributions, we put forward a protocol for model evaluation that goes beyond the evaluation of the epistemic performance of models alone. It also addresses their reactive effects. Indeed, we suggest that a comprehensive analysis of the import of some models—and thereby their appraisal—will, in at least some significant cases, be irreducible to their epistemic import. We label our protocol, which takes these concerns seriously, *contextualist model evaluation*, or *model contextualism* for short.

We analyse an important episode in financial economics, namely the creation of index funds as a consequence of the Capital Asset Pricing Model (CAPM). Index funds have been dubbed by financial economist Burton Malkiel as “unquestionably the most important financial innovation of our time” (Bogle, 2018, pp. xviii–xix). First, we show that a key result of the CAPM is that agents hold the market portfolio, which is composed of all assets traded in the market. The CAPM aptly justified this idea, contradicting what was common practice in the financial industry at the time. Second, we argue that there were two key channels of transmission, which led this model result to be later taken up in the financial industry and eventually led to the creation of the first index fund. This, we argue, is an instance of reactivity: financial markets were not impervious to their being studied by finance scholars.

Studying this historical episode of the CAPM shaping the financial industry motivates our position *model contextualism.* Model contextualism states that model evaluation in terms of a model’s epistemic import is crucial but, in many cases, not enough. Since it is not possible to know a priori whether the results of a model have reactive effects, an adequate appraisal of any model involves, in addition to the epistemic aspect, enquiry into its potential reactive effects. For this, our protocol states that models and their results be analysed according to three criteria: (1) their *epistemic and contextual import*; (2) the specific *channels of transmission* that generate reactive effects of the model in the world; and (3) any *interactions between the epistemic and reactive effects of a model* should be studied. To be a contextualist about model evaluation is to accept all of the three above criteria as a model evaluation protocol.^3^

While the detailed analysis of how the CAPM shaped the financial industry is of interest in and of itself, the primary philosophical contribution of this article is to advance *model contextualism*. The essence of model contextualism is to evaluate models following the above protocol. Model contextualism builds on the traditional model evaluation in philosophy of science that focuses on their epistemic import; on work that considers adequacy for purpose important for model evaluation (Parker, 2020); and on recent work that studies the reactive effects of models in epidemiology, in light of the Covid-19 pandemic (van Basshuysen et al., 2021). It differs from such approaches by insisting that the context is broader than Parker assumes and the success criteria of a model potentially more encompassing than van Basshuysen et al. (2021) assume. We maintain that the evaluation of some models (such as the CAPM) must include its reactive effects (which Parker (2020) does not consider). Moreover, in contrast to both Parker (2020) and van Basshuysen et al. (2021), we maintain that an evaluation of models and their reactive effects is irreducible to their epistemic import alone.

We proceed as follows. In the next section, we introduce the Capital Asset Pricing Model (CAPM). In section three, we describe the features which made the CAPM capable of shaping the financial industry and analyse the context and the conditions that allowed the ideas of the model to travel. In section four, we describe the evaluation protocol. In section five we contrast the protocol with other accounts of model evaluation in the philosophy of science. In section six, we conclude.

## The CAPM

The Capital Asset Pricing Model (CAPM) consists of four core contributions, developed independently by economists Jack Treynor (1962), William Sharpe (1964), John Lintner (1965), and Jan Mossin (1966).^4^ It was the first model to determine the prices and quantities of capital assets in equilibrium under conditions of risk. The objective of the individual investors in the model is to maximise their expected utility by maximising the expected returns of investments subject to risk. The problem to solve is how to allocate their wealth in a portfolio of assets, each of which has different expected returns and risk. The model ultimately captures the relation between the risk an investor is exposed to and the expected return of the investment. In turn, it establishes the kind of risk that determines the differences in returns among assets. As such, the CAPM fully embraces the von Neumann Morgenstern neoclassical paradigm of decision under risk, being a cornerstone framework in financial economics for analysing investor behaviour. Since its inception, the model spawned a large literature on asset pricing models that build from, relate to, or consider the CAPM as a special case.

The main building block of the CAPM is the mean–variance model of Markowitz (1952). Markowitz’s is a normative model, which defines the decision rule that a rational, risk-averse investor should follow in a market in order to choose an efficient portfolio among alternative risky assets. The main insight of this model is that the attractiveness to hold risky assets does not depend on their intrinsic risk alone, as could be perhaps expected, but on all the assets that an investor is willing to hold in their portfolio. This is a consequence of the benefits of diversification that arise by characterising expected return as the mean and the risk faced by investors as the variance, if assets are not perfectly correlated with each other. Markowitz’s decision rule, then, directs investors to buy portfolios of assets on the efficient frontier; portfolios which maximise expected return for a given level of risk. A rational agent maximises their utility when they diversify their investments in an efficient portfolio.

The CAPM describes what the equilibrium conditions would be if all investors in the economy followed Markowitz’s decision rule. The portfolio choices of individual investors represent their particular demands for assets. And, as is standard in economics, by aggregating these individual demands and equating them to asset supplies (which are exogenous to the model), equilibrium prices are determined. The CAPM thus represents individual investors’ behaviour and derives the prices and quantities that must occur in equilibrium.

The CAPM assumes^5^:

1. investors, who maximise expected utility (have mean-variance preferences); prefer higher expected future wealth, and exhibit risk aversion (i.e., prefer lower variance for a given level of expected return);
2. an investment universe consisting of investment opportunities characterised in terms of their expected returns and variances;
3. the availability of a risk-free rate for borrowing and lending;
4. homogeneous investors’ expectations—i.e., all investors have the same beliefs about expected values, variances, and correlations of assets.

The model yielded two crucial results. With respect to the prices of assets, the model implies that asset returns are solely determined by the market risk. This is the risk of investing in the market (as opposed to in riskless assets, such as Treasurys). Furthermore, there is a linear relationship between an asset’s expected return and its quantity of market risk. The quantity of market risk is measured with the market beta, which captures how much the returns on a given asset move together (co-movements) with the market.

With respect to the demands for assets, the model implies that all investors demand and hold the same optimal portfolio of risky assets—they derive the same efficient frontiers, which they hold in proportion to their wealth, depending on their risk appetite. This portfolio is the market portfolio; a portfolio of all the risky assets held by all the agents in the economy.

## Addressing the reactive effects of the CAPM

A way in which we can address the reactive effects of a model is to distinguish two steps. First, to ask whether the model results have the capacity to imply logically the effects that we observe in the world. We will see that the model results of the CAPM have this capacity. Second, to study the channels through which the results of the model disseminate and ultimately end up shaping the world.^6^ We will see that there are two relevant channels that allowed the model results to disseminate. We illustrate each of these steps in the next two subsections.

### The CAPM challenges the financial industry

The results of CAPM about quantities and prices of assets are significant because they contradicted the modus operandi of the investment industry. On the one hand, they implicitly challenged the way investment strategies worked. On the other, they explicitly suggested an alternative for these investment strategies.

There are two aspects of the investment strategies pursued in the financial industry that the CAPM challenged. First, the idea that investment managers had the expertise and knowledge that allowed them to pick stocks that delivered higher returns. There were two “schools of thought” among investment professionals, namely “chartism” and “fundamental analysis” about how to pick winning stock (MacKenzie, 2006a, Chapter 3). “Chartism” involved the analysis of charts drawn from past data, such as the prices transmitted telegraphically using the ticker, in order to predict prices. The idea was that charts made manifest trends and patterns in stock prices that would otherwise be indiscernible. “Fundamental analysis”, by contrast, studied the balance sheets, income statements, and cash flows of a firm to discern their health and prospects and thereby reveal the ‘intrinsic value’ of stock prices. Portfolio managers at Wells Fargo, for instance, were each responsible for over a hundred small trust accounts and worked under the supervision of an investment committee. This committee decided which stocks were acceptable to buy, to hold, or to eliminate from the portfolios. The committee received recommendations from security analysts, who studied and visited the companies they were responsible for (Bernstein, 1993, pp. 235–236).

The CAPM challenged this idea of picking winner stocks because it follows Markowitz’s decision rule of portfolio selection. Given that investors have mean–variance preferences, prefer higher expected future wealth, and exhibit risk aversion, it follows that, in equilibrium agents hold a diversified portfolio. The rationale behind holding a diversified portfolio is Markowitz’ use of the principles of modern probability theory to characterise investment returns as a sequence of random variables. Expected return, then, is the mean and risk the variance. This characterisation leads to a decision rule in which the investor selects portfolios that are on the efficient frontier: maximise expected return subject to a given level of risk or minimise risk given a certain level of expected return. This is the result of the properties of random variables with a normal distribution: the expected value of a weighted sum is the weighted sum of the expected values, but the variance of a weighted sum is not the weighted sum of the variances. There is thus a part of the risk that can be eliminated by diversifying.

By contrast, this doesn’t happen when stocks that are thought to have a higher return are picked. What the strategy of picking stocks misses is that it presupposes that the only risk an investor is exposed to is the intrinsic risk of assets.

The second aspect challenged by the CAPM was that investment strategies were tailor-made for the individual investor, depending on their risk profile and particular circumstances. At Wells Fargo, for instance, the portfolios built for clients typically held about fifteen stocks and tended to be different from each other (1993, Chapter 3). The reasoning was that every client—e.g., individuals, endowment funds, pension funds—had different needs. For instance, investors who wanted to “play safe”, would be advised to invest in value stocks, such as utility companies. These are stocks whose price is low relative to their fundamentals, such as revenues or dividends, and which tend to pay investors regular dividends. Investors who were more daring, by contrast, would be advised to invest in growth stock which, relative to their fundamentals, are expensive. The expectation is that these companies will grow in the future and their true value will be appreciated. But they are riskier and do not provide cash flows, at least in the short term.

From the point of view of the CAPM, the risk profile of the investor is irrelevant for the choice of the portfolio of risky assets; all investors demand the same market portfolio. Risk profiles only determine the proportion of total wealth an investor invests in risky assets. If an investor is risk averse, they invest a small proportion of their wealth in risky assets and the rest in riskless assets, such as Treasurys.^7^ If they are risk loving, they may leverage their position, which is to borrow at the riskless rate of interest and invest their total wealth plus the borrowed portion in risky assets (the market portfolio), taking more risk than the market. If, for instance, this investor is comfortable with the risk level of high-beta stocks, they should not restrict their risky assets to only those high-beta stocks. Instead, the CAPM says they should buy a market portfolio and leverage the position such that the total portfolio has a risk level equal to the high-beta stocks. A portfolio of only the high-beta stocks would deliver lower return than a portfolio that invests total and borrowed wealth in the market portfolio.

This result, which is built into the CAPM, comes from James Tobin’s (1958) “separation theorem”, a proof that the investment decision occurs in two separate, independent steps. One is the process of selecting the efficient portfolio of risky assets and a another is how to divide the total portfolio between risky and riskless assets. Tobin’s was a development over Markowitz’s model in that, in contrast to Markowitz's decision rule, which only contemplated portfolios of risky assets, Tobin acknowledged that individuals with extra cash diversify between some cash (riskless) and risky assets, instead of it being an all or nothing business. Upon introducing this possibility to invest in riskless assets to different degrees, Tobin provided proof that the investment decision of individuals is two-fold.

These were the ideas about the investment industry the CAPM indirectly rejected. With the CAPM, they provided theoretical support to an important stream of empirical research that had been carried out at the Business School of the University of Chicago. There, time series of the stock market, compiled and analysed at the Center for Research in Security Prices (CRSP), based at Chicago under the direction of Prof. James Lorie, showed that stock prices follow a random walk.^8^ This means that price fluctuations over time are statistically independent or that they don’t follow any pattern in particular—there’s no information that can be used from past prices to predict future ones. Sharpe’s CAPM provided a significant theoretical complement to Chicago’s empirical programme. It was a larger theoretical framework that was both in agreement with the findings of the Chicago scholars and, crucially, a result of the theory expected utility previously established by von Neumann and Morgenstern. This theory was considered by economists at the Cowles Commission to provide economics with much sought-after scientific rigour (Herfeld, 2017).

In addition to the implicit rejection of the modus operandi of the financial industry, the CAPM offered an explicit alternative to how investment decisions should be made. This is the result of the model with respect to the demands for assets. This result is that all investors demand and hold a share of the same optimal portfolio of risky assets. The market portfolio is then the sum of these identical portfolios of all individual investors. The proportion in which a given stock is held in the market portfolio is equal to its market value divided by the sum of the market value of all stocks. The reasoning of the market result is this. In equilibrium, all stocks are correctly priced. This means that all stocks yield an expected return that is in accordance with their riskiness and thus no stock is relatively more attractive than any other. The implication is that a rational investor will want to own all stocks. To wit, suppose there is a stock that is not held in the optimal portfolios—i.e., investors do not hold the market portfolio. This means there is no demand for this stock. Its price will thus fall until it reaches a point that makes it attractive for investors to include it in their portfolio. Such price adjustments guarantees that equilibrium is reached and all investors hold all the stocks, thus the market portfolio.

This is, in fact, the rationale behind index funds. Index funds are mutual funds, which replicate the returns of an index, say, the S&P500 by investing in the same assets represented in the index, in the same proportions they have in the index. S&P500 index funds are thought to be close approximations to a market portfolio of the CAPM because they track the performance of the 500 largest companies listed on the US Stock Exchange, which represent between 70 and 80% of the total market capitalisation. Therefore, this index represents (a large portion of) the universe of risky assets that are available in the economy, which investors want to hold.^9^

### Channels of transmission—or, how did the CAPM shape financial markets?

There are many channels of transmission through which the CAPM shaped financial markets. Those who have engaged with the emergence of financial economics as a scientific subdiscipline of economics, have given account of this fact^10^—e.g., Bernstein (1993, 2007), MacKenzie (2006a), Mehrling (2005). In this article we focus on two significant channels through which the CAPM gave impetus to the rise of index funds. Another way to say this is that the story is more complex than we make it here. But we think the two channels we provide are sufficient to make our claim about reactivity plausible.

Perhaps the most important channel is the initiatives that were taken at the Wells Fargo bank in the attempt to bring quantitative analysis to new investment products that the bank could offer. Indeed, Frederick Grauer, Chief Executive Officer of Barclays Global Investors and its predecessors (including Wells Fargo Investment Advisors) from 1983 to 1998, claimed that “[the] CAPM would be only one more model in a long line of models, if it had not impacted commercial practice so dramatically” (Bernstein, 1993, p. 321). The second, related, channel is the work that Jack Treynor (and later Fischer Black) did at Arthur D. Little (ADL), a consultancy firm whose wartime experience in operations research allowed it to offer services in the use of technology to solve computer problems (Mehrling, 2005). The evaluation that ADL did on the investment performance of asset managers contributed to question the expertise and the corresponding fees that active asset managers charged.

#### Wells Fargo

In 1964, John McQuown was hired by Wells Fargo to start the Wells Fargo Management Sciences division. The purpose of this division was to develop quantitative methods for money management. Wells Fargo wanted to develop products that it could sell for beating the market, but to do it independently from the Trust department, the department traditionally in charge of the money management operations (Mehrling, 2005, Chapter 4). At the time, the Trust department operated as much of the rest of the investment management industry: “driven by the views of a few ‘water walkers’ who were supposed to have the magic touch of picking stocks that would outperform” (Mehrling, 2005, p. 103). McQuown, by contrast, would bring rigorous, quantitative research to the Management Sciences Division.

McQuown studied engineering and then did an MBA at Harvard. Afterwards, he worked on Wall Street. While at Harvard, he tried to identify cheap and expensive stocks using a computer programme he developed with a professor from the neighbouring MIT (Ancell, 2012; Bernstein, 1993, Chapter 12). During this time, he also met the Chicago scholars who, like him, had been concerned with the analysis of stock price data bases. Both McQuown and the Chicago scholars had arrived at the conclusion that there are no patterns in the data that can be useful to predict and thus pick stocks. Still, McQuown being part of the industry, was convinced that quantitative analysis was required to satisfy the interests of the clients who trusted portfolio managers with their investments. This is the task he set for himself at Wells Fargo.

In collaboration with the Chicago scholars, McQuown launched two initiatives at Wells Fargo, which contributed to the eventual creation of the first index fund in 1971. One was to raise awareness about the importance of measuring investment performance. Since for McQuown and the Chicago scholars it was clear that there were not patterns in the fluctuations of the prices of stock, it was also clear that any claim to be able to systematically find them was questionable, at best. Measuring investment performance targeted the alleged expertise asset managers claimed to have in finding these patterns and the fees they asked for offering this expertise—the proof of the pudding was in the eating. Wells Fargo thus convinced the Bank Administration Institute, a non-profit corporation whose aims are “to help bank administrators achieve high levels of professional effectiveness and to help solve significant banking problems” (Bank Administration Institute, 1968), to write a report on the measurement of the investment performance of pension funds (Bernstein, 1993, Chapter 12). The report was published in 1968. Chicago professors Lawrence Fischer and James Lorie, as chairman, were members of the advisory committee. Eugene Fama, in collaboration with the advisory committee, wrote a supplement on risk.

In the report, the committee identifies five problems pertaining to the measurement of the investment performance of pension funds. Two are particularly salient: the measurement of the rate of return and the measurement of risk. Indeed, one of the purposes of the report was to convince bankers that measuring risk is a crucial element that had to be incorporated in the measurement of investment performance. Since other measurements of portfolio performance and management contained only a measure of the rate of return, “it is important to indicate why the committee felt so strongly that an additional dimension—risk—was necessary” (Bank Administration Institute, 1968, p. 6). The argument was that, since “one of the most extensively documented propositions in the field of finance is that people can enjoy, on the average, a higher rate of return by assuming more risk” (1968, p. 6), it is important to measure the risk a fund has been exposed to, “to permit a valid comparison of the investment skill exhibited by different fund managers” (1968, p. 6).

In the supplementary chapter written by Fama, “Risk and the evaluation of pension fund portfolio performance”, Fama offers “to give a systematic, logically complete, non-technical discussion of the theory and measurement of risk and their relationship to the evaluation of pension portfolio performance” (Bank Administration Institute, p. 191). This theory he discusses is the CAPM.^11^ Fama describes the CAPM as a tool that would be useful to measure the performance of pension funds. Once the return attributable to risk exposure is factored out, any additional (systematic) return can be used as a measure of the performance of the portfolio manager.

The second initiative was the terms of the collaboration between the Chicago scholars and the Management Sciences division at Wells Fargo. In exchange for the consulting services, Wells Fargo contributed with funding the research of the scholars, including a series of conferences to present work in progress (MacKenzie, 2006a, Chapter 4; Mehrling, 2005, Chapter 3; Wigglesworth, 2021, Chapter 10).

The focus of the first conference, which took place in September 1969 at the University of Rochester, was the empirical test of the CAPM. Here the paper by Black, Jensen, and Scholes, that later in 1972 would be published as “The Capital Asset Pricing Model: Some Empirical Tests”, was discussed. This paper is important because it became the basis for the Stagecoach Fund that Wells Fargo would eventually try to launch and which preceded the first index fund.

In this paper, Black et al. (1972) showed that there was an anomaly in the predictions of the CAPM when the monthly returns of the stock traded in the NYSE between 1931 and 1965 were organised in portfolios according to their market betas. In their test, the low-beta portfolios had higher returns than the CAPM predicted and high-beta portfolios lower returns than the model predicted. While this, strictly speaking, warranted the rejection of the model, their interpretation was instead that there were inefficiencies in the market that could be exploited. Black (1972) in particular, thought that an explanation for the data was that actual markets were suffering from a shortage of credit and introduced a version of the CAPM with restricted borrowing. The original CAPM assumes that investors can borrow freely at the riskless rate of interest. However, in reality investors were apparently not having this possibility.

Instead of challenging the validity of the model, the scholars used this result to suggest to McQuown for Wells Fargo to market a product that would exploit these inefficiencies. They proposed the Stagecoach Fund. “The idea was to invest in low-beta stocks, with what the study by Black, Jensen, and Scholes had suggested was their high return relative to risk, and to use ‘leverage’ (in other words, borrowing) to increase the portfolio’s level of risk to somewhat more than the risk of simply holding the overall market, so also magnifying returns (McQuown interview)” (MacKenzie, 2006a, p. 90).

The Stagecoach Fund didn’t materialise, because there were apprehensions from other members of the Management Sciences division and the Trust department. They argued that the Fund was not properly diversified. In addition, the Glass-Steagall Act, a regulatory framework, presented a number of hurdles that were difficult to overcome in setting up the fund (Black & Scholes, 1974). Wells Fargo therefore abandoned such leverage low-beta fund. Instead, the bank offered its institutional investors a market fund, which was much easier to understand in the context of the CAPM, leaving the decisions to investors about how much wealth they wanted to allocate to this fund. The very first index fund was thus introduced in July 1971 with a six-million-dollar contribution from the pension fund of Samsonite, the luggage manufacturer. The objective of that fund was to track an equally-weighted index of the entire New York Stock Exchange. Subsequently, the bank offered to its institutional investors a market fund whose goal was to track the S&P 500 index. The Samsonite and the S&P 500 funds, are thus, the first implementations of the passive index fund idea motivated by the CAPM.

#### Arthur D. Little

The influence of the CAPM on Arthur D. Little (ADL) came most notably through Jack Treynor, one of the other proponents of the CAPM—in fact, the first one, though his paper wasn’t published until much later. Treynor developed the CAPM from the perspective of the corporate manager who has to decide on the capital investment that delivers the highest future—and thus uncertain—return.

Although Treynor’s interest was in the ex ante problem of deciding which is the investment with the highest return, at ADL the model’s first practical application was to assess the ex post performance of portfolio managers. Since the model gives an estimate of risk for each of the assets in a portfolio and the corresponding expected return, it was easy to use the model as a tool to evaluate portfolio managers’ performance, in the same way in which assessment of pension funds was thought appropriate by McQuown and the Chicago scholars. A proposed measure, nowadays called Treynor’s ratio, adjusts fund returns to the fund’s beta, which is a measure of the fund’s market risk as defined in the CAPM. The underlying rationale was to provide a metric that would allow to distinguish those managers who added value from those that did not.

A disappointing performance of mutual funds and pension funds managers relative to the high sales commissions and management fees charges, drew the attention of the Security Exchange Commission (SEC). The SEC had proposed the Investment Company Amendments Act of 1967 to impose regulatory control on these charges. In response, the Investment Company Institute (ICI) hired ADL to use Treynor’s ratio to demonstrate superior performance of active mutual funds and thus argue against the regulatory control.

In their report, ADL’s consultants, mainly led by Bob Fahley and Fischer Black, relied not only on the Treynor’s ratio but also on two other CAPM-based performance measures: Jensen (1968) and Sharpe (1966). Jensen uses alpha as the difference in returns of a mutual fund corrected for market risk and those of a passive market index. According to the CAPM, the alpha should be zero given that the returns are solely determined be the exposure to market risk. Any positive alpha would thus indicate a superior performance. Sharpe offered the Sharpe ratio, which divides the excess returns of a fund, i.e., the reward the fund brings above the return on the riskless asset, by its total risk. It thus measures the reward that a fund brings per unit of risk. According to the CAPM, the total risk is an appropriate measure for well-diversified portfolios which mutual fund managers are supposed to hold. Again, any positive number of this ratio would indicate a superior performance.

The results were, however, disappointing for the mutual fund industry. Most of the evidence suggested that active asset managers were performing poorly and were not able to outperform the passive market index. This suggested that professional asset managers did not add value and investors might as well buy and hold the entire market portfolio. Ultimately, these results were included only in the committee files and were not discussed at the public hearings, but ADL’s research and consequently their inability to defend the mutual fund industry, raised awareness about the importance of evaluating the performance of the professional asset managers and, most importantly, about their inability to outperform the passive market index.^12^

This contributed to the work of the Advisory Committee on Endowment Management, commissioned by the Ford Foundation, which found that university and college endowments performed even worse than mutual fund industry. These widespread results gave sufficient impetus for the industry to rethink their commitment to actively managed funds to consider developing passive investments funds.

## Model contextualism

### Introducing model contextualism: a protocol for model evaluation

Above we have given an account of reactivity. The CAPM, used to describe the workings of capital markets in the framework of neoclassical economics, was used by two institutions, the Wells Fargo bank and Arthur D. Little, to buttress their distinctive quantitative approaches, eventually leading to the creation of the first index fund. Traditionally, the philosophical appraisal of this model would be in terms of its epistemic import. More specifically, in terms of its capacity to elucidate aspects of its target, namely the differences across the expected returns of capital assets. But, can the reactive effect of this model, the creation of the first index fund, be accounted for in such an epistemic appraisal? The possibility to account for and appraise this reactive effect is what motivates our protocol *model contextualism*. In the remainder of the article, we introduce the protocol and compare it to other contributions to model evaluation.

As stated at the outset, model contextualism states that models and their results be analysed according to three criteria: (1) their *epistemic and contextual import*, (2) the specific *channels of transmission* that generate reactive effects of the model in the world and (3) any *interactions between the epistemic and reactive effects of a model.*

We will first explain how the analysis of the historical episode can be generalised to a protocol for model evaluation that captures both the epistemic and reactive import of a given model. Second, we will contrast model contextualism with recent approaches to model evaluation put forward in Parker (2020) and van Basshuysen et al. (2021).

### Model contextualism and the CAPM case

We discuss the three criteria of the model evaluation protocol in terms of the CAPM case that has motivated it.

#### The epistemic and contextual import of the model results

We take the results of the model to be the logical implications of the model that are considered significant or relevant according to the developers of the model. As we discussed above, for the CAPM these were the results with respect to the prices and quantities of assets that would be demanded in equilibrium. Appended to these are the significance of beta as a measure of the quantity of market risk and the linear relationship between market risk and expected returns.

The evaluation of the epistemic import of these results may take two forms. First, the “representational” evaluation of the theoretical model, which involves determining the model’s capacity to elucidate aspects of its target—for instance, to determine whether the model identifies stable and, potentially, robust relations that may be useful for causal claims or counterfactual inferences that, in turn, may be considered explanatory.^13^ This is the kind of evaluation that has been favoured by the philosophical literature on models. Second, the empirical evaluation of the model, which involves confronting the empirical counterpart of the theoretical model with the available data. This form is exemplified by the tests of the CAPM carried out by financial economists as an adequate equilibrium theory of capital markets. The tests, such as Black et al. (1972), tested the linear relationship between market risk and expected returns, with the purpose to determine whether the model accurately describes cross-sectional differences in expected returns.^14^

The detailed exposition of our case illustrates that it is not enough to stop at this purely epistemic assessment which confronts the model with its target there’s also the assessment of the contextual import of the model results. This refers to the significance of the results *in the context in which they were obtained.* Put differently, while the evaluation of epistemic import can be addressed anachronistically (and often is), the assessment of the contextual import cannot––its significance is with respect to the context in which these model results were obtained. As we argued in section two, the CAPM model results contributed to challenging the status quo of the investment industry with respect to the way investment strategies worked and suggested an alternative for these investment strategies. There are at least two aspects of the contextual import that are particularly salient for our case.

First, the specific questions that were pursued by the modellers as well as their motivations for pursuing them and the interpretation of the results.^15^ This aspect is salient because it makes manifest the relevance of how the test of the CAPM carried out by Black et al. (1972) was interpreted and the consequences this had for the creation of the first index fund. As discussed above (pp. 10–11), the Chicago consultants working for Wells Fargo did not interpret Black et al. (1972) as a failed test that warranted the rejection of the model—and which potentially would lead a purely epistemic assessment to be regarded as an epistemic failure—but rather as a signal of the presence of inefficiencies in stock valuations in financial markets; for them the world was imperfect, not the model. This interpretation is what led them to propose the Stagecoach Fund, which preceded the first index fund.

Second, analysing the contextual import involves understanding the ways in which the model in question is related to other empirical and theoretical models, which in turn allows to appreciate the significance of model results *relative to* cognate models (Vergara-Fernández et al., 2023). This relative significance is particularly important because, in the case of the CAPM, it is what allowed us to determine that it was the CAPM—and not Tobin’s or Markowitz’s models, on which the CAPM builds—which gave impetus to the index funds. In other words, because we know what the ‘added value’ of the CAPM is in relation to these cognate models on which it builds, we can single out the CAPM as responsible for challenging the status quo of the financial industry, even if these other models also did—recall that the challenge to the idea to tailor investment strategies to the client’s needs was based on Tobin’s separation theorem.

#### Channels of transmission

Identifying the *channels of transmission* goes beyond merely establishing *that* the model may have reactive effects. It involves linking aspects of the results of the model that are significant, with the way in which these results are disseminated and used by interested parties—e.g., individuals and institutions—giving impetus to the reactive effects.

Settling on unequivocal channels of transmission is no easy task. In section two we discussed the role of Wells Fargo and ADL as institutions that, for different reasons, established bridges between academia and the industry, as well as the role of key figures within these organisations. But, as we stated above, the story of how the CAPM gave impetus to index funds is much more complex than we suggest, in at least two ways. There are other factors that also contributed to rise of index funds—e.g., the market crash of 1974—and the historical accounts on which we rely do not always give the same significance to specific events.

What is clear, however, is that, in order to identify these channels of transmission, it was necessary to rely on historical work and, in particular, to be able to pick out the significance of institutions and characters from larger narratives. This significance comes from recent developments in the history of economics such as Cherrier (2014), Cherrier & Saïdi (2021) and Emmett (2011), which have highlighted the importance of institutions, conferences, and seminars in disseminating ideas and thereby shaping economics as a scientific discipline. This work has made these forms of dissemination of ideas a legitimate object of study for historians of economics. Thus, we suggest, the history of economics is an indispensable source for identifying channels of transmission. More generally, such historical analysis is crucial for identifying channels of transmission of reactive effects of models and thus a comprehensive appraisal of any scientific model.

#### Interactions between epistemic and reactive effects

The aforementioned analysis raises the question of what to make of potential (3) *interactions between the epistemic import and the reactive effects of a model*. We do not present a fully worked out analytic framework for these interactions—mainly because we do not believe that such complexities can be analysed a priori. Still, there are some aspects of such interactions that we can highlight.

As we have demonstrated, there is a context that has been “made” by the model: first index funds were created and later passive investment more generally has become a significant investment strategy. So far, the reactive effect of the CAPM.

An important question about possible interactions between epistemic import and reactive effects of the model is whether the (changed) world has somehow had a feedback effect back on the model. In a nutshell, is there the converse phenomenon of the “context making the model” in the CAPM case? We think the answer is affirmative, in the following sense. Take the market beta in the CAPM. The beta in the theoretical CAPM is defined with regards to all risky assets in the economy. Thus, the market portfolio implied by the CAPM is meant to include not only the stock market but all other assets as well. Indeed, early academic tests of the CAPM estimated beta with respect to the market portfolio measure that included the stock market index but also indices of other assets such as bonds or real estate (Stambaugh, 1982; Shanken, 1987). In practice, however, in the financial industry, beta is not quite as defined in the model. It has an empirical extension in the world (partly in virtue of creating it, and partly in virtue of the world filling in the meaning of this term). The existence and burgeoning popularity of index funds as an investment vehicle that allows investors to gain exposure to the market risk, contributed to the shift in the interpretation of the market portfolios towards the stock market index. In turn, the beta is now often understood with regards to stock market indices alone. In fact, many of the extensions to the CAPM in academia, most notably the multifactor models, focus exclusively on the stock market-based measure of the market portfolio. And the analysis of capital markets has extended to other stock market-related variables rather than attempt to capture the market portfolio as defined in the original CAPM. This is how the context that was created by the model now influences the interpretation of the terms in the model.

And so, the context makes the model. It may well be the case that the way in which the context has made the model may be at odds with how the model was conceived of theoretically (and was conceived of for epistemic gains). So, if the context is “making the model” in this way, then it might influence, even potentially undermine, the epistemic performance of the model. Or we might conclude there are now two different models, the original one, and the one that was shaped by the world.

Analysing the interactions between the epistemic import and the reactive effects of a model is a challenging and complex task. We do not wish to suggest that the epistemic import and reactive effects can always be completely separated. And yet, following the model evaluation protocol gives analytic tools that help telling apart many different kinds of aspects of model evaluation – in as far as that is possible. Doing so strengthens the analyst’s capacity to do so.

## Model contextualism and other accounts of model evaluation

We now discuss how model contextualism moves forward the model evaluation literature.

There are recent contributions that emphasise different aspects of the reactivity (or performativity) of models. Boldyrev and Ushakov (2016) argue that models can be built with the purpose of shaping the world and that this ought to be accounted for in our philosophical accounts of models: “We would propose to complement representational and non-representational accounts by looking at the ways economic modeling is aimed at transforming its own target.” (p. 41). By the same token, Tee (2019) argues against the traditional view in philosophy of modelling to take models as having only a “passive epistemic role”. Tee suggests that, in addition to the traditional criterion of model-world representation, at least some models could be evaluated by their “constructive” capacity. We go a step further than these contributions by trying to elucidate how appraising models would or could be like if this feature of models were taken into account. Thus, *model contextualism* integrates the demand for taking into account the reactivity of models into a comprehensive account of model evaluation.

In pursuing a comprehensive approach to model evaluation, *model contextualism* partly relies on received approaches to evaluating models epistemically in the philosophy of science, focusing on the representational and explanatory import of models. How is it different from approaches in this literature? Just as these received approaches, model contextualism acknowledges the importance of epistemic evaluation. However, model contextualism seeks to evaluate models more comprehensively, including the potential reactive effects models may have.

Our approach differs from others in three ways. First, take the relevance of the contextual import for model evaluation. Most philosophers now agree on the importance of use and purpose of models as defined by modellers and other users. These have long been recognised as being part and parcel of modelling exercises—e.g. Giere (2004), Mäki (2009a, 2009b, 2017). Mäki (2009b, 2017), in particular, who has gradually modified his account of models to include aspects related to the practice suggests: “The modellers’ goals and contexts provide the *pragmatic constraints* on models.” (Mäki, 2009a, 2009b, p. 179). And yet in cases such as the CAPM, the context was not just a constraint. The context was shaped by the model, too. Our approach does not just *acknowledge* the context in which models are developed but stresses its significance for model evaluation. Our labelling the evaluation protocol “contextualist” is meant precisely to emphasise the importance of the context for model evaluation.

Second, take the “scope” of the relevant contextual aspects for model evaluation. The recent “adequacy-for-purpose” view by Parker (2020) proposes to evaluate models by making precise the *purpose* of models with respect to which their adequacy is evaluated. Accounting for the purpose for evaluation is indeed a way to take the context of a model seriously. However, Parker approaches model evaluation mainly from an epistemic perspective. While the account acknowledges that some purposes of models may be practical, it is assumed that, even in these cases, “the intended contribution of the model is often epistemic […]” (2020, p. 460), thus reducing the relevant aims to *epistemic* aims*.* These centre on notions of epistemic success and reliability. That is to say, there is not sufficient room in this approach for capturing the kind of change in the world that we have seen in the CAPM case. Consider Parker’s definition of adequacy: “in general terms what is required is that the model stands in a suitable relationship with a target, (type of) user, (type of) methodology, (type of) circumstances, and purpose jointly. Put differently, the model must constitute a ‘solution’ in a kind of problem space.” (Parker, 2020, p. 475) Now, on a first reading of this quotation one might think that it sounds not particularly geared towards the epistemic. The definition mentions many different elements that are relevant for model evaluation. And so, might these be employed for analysing how a model shapes the world? To attempt to do so, one might try to capture the reactive effects of a model by its adequacy-for-purpose of a model in terms of a *type* or *context of use* (Parker, 2020, p. 462). However, the types and contexts that are cited by Parker are entirely scientific, or cases of applying science; they don’t venture into the *context* being shaped in unintended and more fundamental and ways like we have seen in the CAPM case. Parker’s approach is geared towards analysing how the epistemic import of a model plays out in a context: how it is epistemically adequate-for-purpose for a (type of) user, (type of) methodology, and (type of) circumstances.

Finally, the approach pursued by van Basshuysen et al. (2021) is perhaps closest in spirit to *model contextualism*. They analyse the performativity of epidemiological models in the Covid-19 pandemic. They start with characterising predictive success as the most important epistemic desideratum for evaluating epidemiological models. In similar vein to our approach, they also aim to make precise how exactly these models were performative. They go on to identify three different performative effects of epidemiological models: how the models changed (a) their own predictions, (b) the policy advice, and (c) individual responses to the pandemic. Moreover, they conclude: “we thus suggest that both predictive and performative capabilities should be considered side-by-side when appraising […] models.” (van Basshuysen et al., 2021, p. 121). This conclusion echoes the aim of *model contextualism*: to analyse both the epistemic import of the model results and their reactive effects.

At the same time, there are differences with *model contextualism*: for one, our model evaluation protocol is purposefully non-committal with regards to the epistemic import of the model results. Being open about this is important for evaluating models whose epistemic contribution is contested (like it is for the CAPM, as suggested by the failed empirical tests; see Vergara-Fernández et al. (2023)). In contrast, van Basshuysen et al. (2021) narrowly assume ‘predictive capabilities’ as a single epistemic desideratum (and success criterion) for the epidemiological models they evaluate.^16^ They also go on to evaluate the performative capabilities and effects in terms of narrowly defined success criteria of the epidemiological model (how the performativity impacted the predictions, the policy advice, and individual responses to the pandemic).^17^ In contrast, *model contextualism*—while demanding about establishing transmission channels that make precise how model results can and do have reactive effects through careful historical study—is again purposefully open and non-committal about model success.

### Model contextualism in perspective

We have defended *model contextualism—*a model evaluation protocol that tells the evaluator to address all three criteria in the manner demonstrated for the CAPM case. We have also shown how other prominent accounts in the literature can be subsumed by (some steps of) the model evaluation protocol. For instance, one might want to evaluate models without analysing the performative effects in detail (so, no step 2 or 3 of our protocol), but still acknowledge implications for the context, such as that models are used for policy advice, or that modellers are taking directions/requests from model users (e.g., Parker, 2020). And, indeed, one might also want to adopt the idea of ‘priority of the epistemic import’ in one’s evaluation. For instance, Parker (2020) does so in her particularly mild variant of model contextualism that only completes a weak interpretation of what constitutes step (1) of the protocol. And while van Basshuysen et al. (2021) offer a comprehensive analysis of both epistemic and reactive import of epidemiological results, they also do so firmly prioritising the predictive import of the models in question.

And so, model contextualism pursues a perspective that learns and differs from all the aforementioned approaches. We focus on the implications that reactivity has for the way in which we *appraise scientific models*, especially in financial economics. In that sense, we take the aforementioned analyses as sufficient for taking for granted that it is possible for theories to shape the world. Model contextualism is thus less concerned with the question of *if* or *whether* models such as the CAPM shape the world, but more on *how* they do so, and how their shaping the world is rooted in (and potentially at odds with) their epistemic performance.

Our analysis also entails two challenges. The first challenge is methodological, for the philosophy of modelling. *Model contextualism* has significant capabilities to analyse the reactive effects of models. At the same time, it is also demanding in terms of the descriptive and historical accuracy required for the analysis. It thus challenges the philosophy of modelling to adopt a historically informed approach in order to go beyond the epistemic evaluation of models. Having said that, analysing the reactive effects of modelling, and in particular how they interact with the epistemic import remains challenging. Our analysis piggybacks on the historical evidence available but, of course, in the absence of this evidence—say, because the model in question is a particularly novel one—it would involve that the philosopher has to become a historian and a sociologist, too. Perhaps more positively then, the suggestion would be that thorough model evaluation, though primarily of philosophical interest, should be done in conjunction with historians and sociologists of science.

The second challenge is normative, for the philosophy of science of finance. Given the reactivity of their models, should finance scholars factor in any “reactive risk” that they might be able to detect in their analyses? For the philosophy of science of finance, this question creates the need to determine the right standards with which to evaluate the responsibility of financial scholars in this regard. This question has received attention in the recent literature on the reactivity of epidemiological models (such as van Basshuysen et al., 2021 and Winsberg & Harvard, 2022). For the philosophy of science of finance, this question is perhaps even more complex: market events such as financial crises are perhaps even harder to delineate for analysis (even ex post) than pandemics. The epistemic desiderata for models in finance are also less settled than, for instance, those in epidemiology.

## Conclusions

Our analysis yields two types of result. First, our CAPM case study is of both historical and current interest in and of itself, as it helps to appreciate the genealogy of index funds and passive investing. Furthermore, it demonstrates what kind of *contextual import* was at work in which kind of *transmission channels* to shape index fund investment. More generally, our case study shows the relevance of reactivity (or “performativity”) for financial models. This will be particularly fruitful for currently emerging phenomena with similar dynamics. There has been a recent rise in “factor models” in academic financial research, and these factors are increasingly exploited in investments on financial markets. An analysis of the reactive effects of *these* models with a contextualist perspective will also be fruitful.

Second, the analysis of the CAPM case has motivated *model contextualism*, an approach to model evaluation that combines important motivations of both epistemic model evaluation in the philosophy of science and analyses of “performativity” in the sociology of science literature. *Model contextualism* goes further than epistemic and “adequacy-for-purpose” kinds of model evaluations, as it takes the context more seriously than these approaches: it is also capable of elucidating the *reactivity* of models, and it does not reduce their analysis to the epistemic dimension.

## Footnotes

1 To be sure, the phenomenon of science shaping its object of study has been analysed by philosophers. Hacking (1996) is a classic example, having significant impact on the literature on social ontology. More recently, sometimes dubbing it reactivity, scholars have discussed the phenomenon regarding other aspects of scientific practice: Vesterinen (2021) investigates ‘looping effects’ in explanation, Jiménez-Buedo (2021; Jiménez-Buedo & Guala, 2016) analyse reactivity in economic experiments, and Runhardt (2021)) analyses reactivity in measurement.

2 MacKenzie’s project is to offer detailed evidence in support of the claim that models *are* performative, so “an engine, not a camera”. We build on his work in the sense that he initiated the discussion about the performativity of financial models and offered a rich history to support his case. Our project, by contrast, is to offer an account of model appraisal which considers this performative aspect, using the CAPM and elucidating the conditions that made this performative role possible.

3 Our analysis is thus *contextualist* (and not merely contextual). We will argue that *model contextualism* in this sense goes much further than the widely accepted acknowledgement that “contextual” factors, such as the purpose of models as defined by modellers and other users, matter (Giere (2004); Mäki (2009a, 2009b, 2017)).

4 Jack Treynor’s (1962) derivation is an unpublished manuscript (published in 1999 in a book). Sharpe, however, was aware of it and acknowledged the similarity of their approaches in his (1964).

5 Here we rely on Sharpe’s assumptions. Other versions of the model have slightly different assumptions.

6 In analysing these mechanisms, we build on and go beyond MacKenzie, who does suggest that the CAPM was performative in bringing about index funds: e.g. “Among the consequences of the growth of index funds and of covert index tracking was that the Capital Asset Pricing Model’s prediction that all investors would hold the same portfolio of risky assets gradually became less false than it had been when the model was formulated in the early 1960s” (p. 87). More recently, Braun (2016) has complemented the story, suggesting that while the CAPM was pivotal in bringing about index funds, the emergence in the nineties of Exchange Traded Funds (ETFs) made them particularly significant as a share of global assets under management.

7 Strictly speaking, Treasurys are not completely riskless. But they are assumed to be so relative to the alternatives; the United States government debt is—as of now—the safest asset available.

8 Eugene Fama (1965, 1970) built on this research to formulate the famous Efficient Market Hypothesis (EMH).

9 There are also other indexes that track more market capitalisations, and also of other countries. These other indexes can be seen as instantiations of the CAPM market portfolio.

10 There are also recent popular accounts of the rise of index funds such as Bogle (2018) and Wigglesworth (2021) which offer a rich amount of detail to this fascinating story. Interestingly, in his book Bogle claims to have established the first retail index fund at Vanguard purely as a solution to the corporate governance problems facing Wellington Investment and allegedly not ever having heard of the Chicago scholars. This is highly implausible: Bogle attended the conferences sponsored by Wells Fargo discussed in this article.

11 Wigglesworth (2021, p. 80) reports that, in the summer of 1972, the Institutional Investor Magazine dubbed Wells Fargo “The Temple of Beta”, alluding to its increasing reliance on the CAPM.

12 In the same hearings, Paul Samuelson made a case for the regulations arguing that the mutual fund industry was not competitive enough and that the performance of the funds was indistinguishable from the performance of stocks selected at random.

13 We call this form “representational” because philosophers of science tend to agree that the epistemic import of theoretical models is rooted in their representational capacity. There are some exceptions, like Grüne-Yanoff (2013) and Knuuttila (2005) but their stance on representation is not at odds with this appraisal of theoretical models.

14 We discuss these tests and their implications for the field in Vergara-Fernández et al. (2023).

15 See Vergara-Fernández & de Bruin (2021) for an account of how epistemic and non-epistemic values motivated the Modigliani and Miller propositions, another cornerstone model in financial economics.

16 This choice strikes us entirely reasonable for the models at hand in the analysis of van Basshuysen et al (2021).

17 Again, this choice is entirely reasonable for the models at hand in the analysis of van Basshuysen et al (2021).
